# Ventricular arrhythmic burden before and after mitral valve repair in mitral valve prolapse: the role of mitral annular disjunction

**DOI:** 10.3389/fcvm.2026.1889270

**Published:** 2026-10-07

**Authors:** Ümeyir Savur, Berhan Keskin, Aysel Akhundova, Aykun Hakgor, Bilal Boztosun

**Affiliations:** Department of Cardiology, Istanbul Medipol University, Medipol Mega University Hospital, Istanbul, Türkiye

**Keywords:** mitral annular disjunction, mitral valve prolapse, mitral valve repair, premature ventricular complexes, risk stratification, ventricular arrhythmia

## Abstract

**Background:**

Mitral valve prolapse (MVP) is increasingly recognized as an arrhythmogenic condition, with a subset of patients exhibiting ventricular arrhythmias beyond the hemodynamic effects of mitral regurgitation (MR). Mitral annular disjunction (MAD) has been proposed as a structural marker associated with arrhythmic risk, but its relationship with postoperative ventricular arrhythmias after mitral valve repair remains incompletely understood. We aimed to evaluate changes in ventricular arrhythmic burden following mitral valve repair and to investigate the association between preoperative MAD and postoperative ventricular ectopy.

**Methods:**

This retrospective observational study included 77 patients with MVP who underwent surgical mitral valve repair for severe MR and had both preoperative and postoperative 24-h Holter recordings. Premature ventricular complex (PVC) burden and ventricular arrhythmias were assessed before and after surgery. MAD was evaluated retrospectively using transthoracic echocardiography. Multivariable linear regression, sensitivity analyses, and sequential model comparison were performed to evaluate the independent and incremental association of MAD with postoperative PVC burden.

**Results:**

Overall PVC burden decreased significantly after mitral valve repair. Patients with preoperative MAD had a substantially higher PVC burden both before surgery [7,800 (7,000–9,575) vs. 4,200 (3,200–6,200) PVCs/24 h, *p* < 0.001] and after surgery [5,700 (5,175–6,425) vs. 2,200 (1,800–3,500) PVCs/24 h, *p* < 0.001] than those without MAD. The relative reduction in PVC burden was comparable between groups (−35.4 ± 20.6% vs. −39.5 ± 29.5%, *p* = 0.113). In multivariable analysis, preoperative MAD remained independently associated with higher postoperative PVC burden after adjustment for clinical covariates, prolapse phenotype, residual mitral regurgitation, and baseline PVC burden.

**Conclusions:**

Mitral valve repair was associated with a significant reduction in ventricular ectopic burden in patients with MVP. However, preoperative MAD identified patients with a higher postoperative ventricular ectopic burden despite a similar proportional reduction in PVCs. These findings support the potential value of postoperative rhythm surveillance in patients with preoperative MAD.

## 1 Introduction

Mitral valve prolapse (MVP) is a common valvular abnormality, affecting approximately 2%–3% of the general population, and represents a leading cause of primary mitral regurgitation (MR) in developed countries (1). The underlying etiologies include Barlow's disease, fibroelastic deficiency, and mixed degenerative forms (2). Although the overall prognosis is often favorable, up to one-quarter of patients may develop progressive MR, potentially leading to left ventricular remodeling, heart failure, and increased mortality (3). Beyond its hemodynamic consequences, a subset of patients independent of MR severity exhibits a more arrhythmogenic phenotype, characterized by an increased risk of ventricular arrhythmias (VAs) and sudden cardiac death (SCD) (4). Reported arrhythmic features include bileaflet prolapse, Barlow's disease, mitral annular disjunction (MAD) (5, 6), frequent premature ventricular complexes (PVCs), nonsustained ventricular tachycardia (NSVT), T-wave inversion in inferior leads, and late gadolinium enhancement (LGE) on cardiac magnetic resonance imaging (CMR) (7, 8).

MAD, traditionally defined as systolic separation between the atrial wall-mitral valve junction and the underlying ventricular myocardium, is increasingly recognized as a heterogeneous anatomical finding rather than a uniformly pathological entity. Contemporary multimodality imaging studies have demonstrated that limited anterior and inferior disjunction are frequently observed in structurally normal hearts, whereas inferolateral disjunction appears uncommon and is the phenotype consistently associated with mitral valve prolapse, myocardial fibrosis, systolic curling, ventricular arrhythmias, and adverse clinical outcomes (9–11). Accordingly, the arrhythmogenic significance of MAD is now considered to depend on its anatomical location, circumferential extent, and accompanying structural abnormalities rather than its mere presence. However, the extent to which this structural abnormality influences arrhythmic outcomes following surgical correction of MR remains incompletely understood.

Mitral valve repair is the preferred treatment for severe MR, offering favorable long-term outcomes, including improved survival, preservation of left ventricular function, and avoidance of prosthesis-related complications compared with valve replacement (12). While surgical correction effectively addresses the hemodynamic consequences of MR, its impact on ventricular arrhythmic burden, particularly in patients with underlying structural substrates such as MAD, remains uncertain.

In this context, the present study aimed to evaluate changes in ventricular arrhythmic burden before and after mitral valve repair using 24-h Holter monitoring, and to explore the potential association between MAD and pre- and post-operative arrhythmic activity in patients with MVP.

## 2 Methods

### 2.1 Study design and population

This retrospective observational study included patients who underwent surgical mitral valve repair for severe mitral regurgitation secondary to mitral valve prolapse at Istanbul Medipol University Hospital between January 2018 and August 2022 and fulfilled the predefined eligibility criteria (Figure 1). The total number of degenerative mitral valve repair procedures performed at our institution during the study period could not be reliably retrieved from the available institutional records; therefore, the study cohort should not be considered a consecutive series of all mitral valve repairs performed during this period. Eligibility for the present study required the availability of both preoperative and postoperative 24-h Holter monitoring recordings together with complete clinical and echocardiographic data. Clinical characteristics, echocardiographic parameters, and Holter monitoring data were obtained from the institutional electronic medical records.

**Figure 1:**
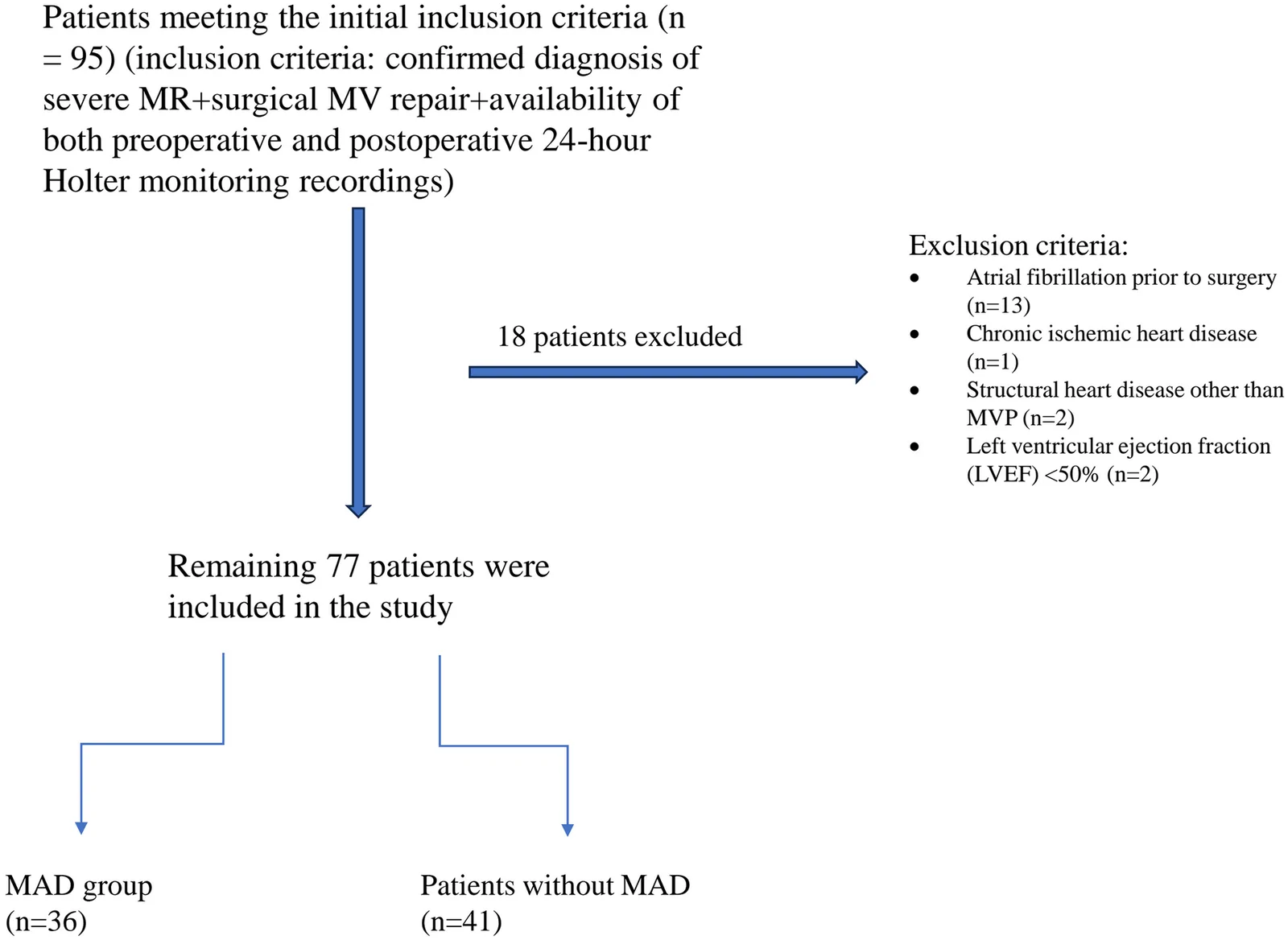
Study flow diagram.

#### 2.1.1 Inclusion and exclusion criteria

Figure 1 Patients were eligible for inclusion if they met the following criteria:

1. confirmed diagnosis of MVP with severe MR,
2. undergoing surgical mitral valve repair, and
3. availability of both preoperative and postoperative 24-h Holter monitoring recordings.

Patients were excluded if they had:

1. atrial fibrillation (AF) prior to surgery,
2. chronic ischemic heart disease,
3. structural heart disease other than MVP, or
4. left ventricular ejection fraction (LVEF) < 50%.

### 2.2 Echocardiographic assessment

Mitral valve prolapse was diagnosed according to current guideline criteria as systolic displacement of one or both mitral valve leaflets ≥2 mm beyond the mitral annular plane into the left atrium in the parasternal long-axis view (13).

The severity of mitral regurgitation was evaluated using an integrative multiparametric approach, in accordance with current American Society of Echocardiography and European Association of Cardiovascular Imaging recommendations, including assessment of vena contracta width, effective regurgitant orifice area, and regurgitant volume (14).

Mitral annular disjunction was assessed retrospectively from stored transthoracic echocardiographic images obtained in the parasternal long-axis view at end-systole, when the separation between the mitral annulus and the ventricular myocardium is most pronounced. MAD was defined as the maximal distance between the atrial wall-mitral valve junction and the basal left ventricular myocardium (15). Measurements were performed using two-dimensional transthoracic echocardiography, and the presence of MAD was categorized dichotomously as present or absent for subgroup analyses. All measurements were performed offline by a single experienced echocardiographer who was blinded to the Holter monitoring results. To minimize measurement variability, all assessments were conducted according to predefined criteria and under standardized analysis conditions.

Because the study was retrospective, MAD assessment was limited to two-dimensional parasternal long-axis transthoracic echocardiography. Consequently, circumferential localization and extent of annular disjunction could not be reliably assessed, and differentiation between common anatomical variants and the inferolateral arrhythmogenic phenotype described in recent multimodality imaging studies was not possible.

### 2.3 Holter monitoring and arrhythmia analysis

Holter recordings were retrieved retrospectively from the hospital's digital archive. Preoperative Holter monitoring was performed as part of the routine pre-surgical evaluation. Postoperative Holter recordings were included in the analysis only if obtained at least 6 months after mitral valve repair, allowing sufficient time for hemodynamic stabilization following surgical correction. When multiple postoperative Holter recordings were available, only recordings performed within the predefined 6–12-month follow-up interval were considered eligible for analysis. Among these, the Holter recording obtained closest to the 6-month follow-up time point was selected for the final analysis.

All Holter recordings were reassessed by a cardiologist experienced in arrhythmia evaluation using a standardized institutional reporting protocol. For each 24-h Holter recording, the total number of PVCs and the presence of nonsustained or sustained ventricular tachycardia (VT) were documented. Premature ventricular contraction (PVC) burden was expressed as the total number of PVCs per 24 h.

Information regarding rhythm-related medical therapy at the time of Holter monitoring was retrieved from the available electronic medical records, including beta-blockers, non-dihydropyridine calcium channel blockers, and class I/III anti-arrhythmic agents. In the study cohort, beta-blockers were the only rhythm-related medications used, whereas no patients were receiving amiodarone, sotalol, flecainide, propafenone, diltiazem, or verapamil at the time of Holter evaluation. Although the retrospective nature of the study may have introduced minor variability in documentation, standardized review criteria were applied to ensure consistency and minimize subjectivity.

### 2.4 Statistical analysis

Continuous variables were assessed for normality using visual inspection and the Shapiro–Wilk test. Given the skewed distribution of PVC burden, PVC-related variables were log-transformed using the natural logarithm of (1 + PVC count) prior to regression analyses. Continuous variables are presented as mean ± standard deviation (SD) or median [interquartile range (IQR)], as appropriate according to their distribution, while categorical variables are expressed as counts and percentages. Between-group comparisons (MAD vs. no MAD) were performed using the independent-samples *t*-test for normally distributed continuous variables and the Mann–Whitney *U* test for non-normally distributed continuous variables. Categorical variables were compared using the chi-square test or Fisher's exact test, as appropriate.

Pre- to postoperative changes in PVC burden were assessed using the Wilcoxon signed-rank test for paired observations. Median paired changes and their 95% confidence intervals were estimated using percentile bootstrap resampling (20,000 iterations). Between-group differences in change were evaluated using the Mann–Whitney *U* test, with Hodges–Lehmann location-shift estimates and bootstrap 95% confidence intervals reported to quantify the magnitude of between-group differences.

To directly assess whether the longitudinal change in PVC burden differed according to MAD status, pre- and postoperative PVC measurements were additionally analyzed in a patient-level repeated-measures model. Because of the skewed distribution of PVC counts, PVC burden was transformed as ln(1 + PVCs/24 h). Time (preoperative vs. postoperative), MAD status, and their interaction (time × MAD) were included as fixed effects, with a patient-specific random intercept to account for within-patient correlation. The time × MAD interaction was used as the primary test of whether the magnitude of change differed according to MAD status. To directly assess whether the longitudinal change in PVC burden differed according to MAD status, pre- and postoperative PVC measurements were additionally analyzed in a patient-level repeated-measures model. Because of the skewed distribution of PVC counts, PVC burden was transformed as ln(1 + PVCs/24 h). Time (preoperative vs. postoperative), MAD status, and their interaction (time × MAD) were included as fixed effects, with a patient-specific random intercept to account for within-patient correlation. The time × MAD interaction was used as the primary test of whether the magnitude of change differed according to MAD status. In a sensitivity analysis addressing potential confounding by residual mitral regurgitation and beta-blocker treatment, the analysis was restricted to patients with successful repair, defined as residual MR less than moderate, and beta-blocker use was included as a time-varying covariate. Generalized estimating equations with patient-level clustering and an exchangeable working correlation structure were used for this sensitivity analysis.

Univariate linear regression analyses were performed to identify variables associated with pre-operative and post-operative PVC burden. Variables with clinical relevance and/or statistical significance (*p* < 0.05) in univariate analysis were entered into multivariable linear regression models. The primary multivariable model evaluating determinants of post-operative PVC burden included log-transformed pre-operative PVC burden and MAD, along with selected clinical and echocardiographic covariates. To assess the relative contributions of MAD and baseline arrhythmic burden, sensitivity analyses were conducted using two additional models: one excluding pre-operative PVC burden and another excluding MAD. This approach was used to evaluate potential collinearity and to better understand the mechanistic relationship between structural substrate and arrhythmic burden. All regression models were fitted using ordinary least squares with heteroscedasticity-robust standard errors (HC3). Multicollinearity was assessed using variance inflation factors (VIF). Model performance was evaluated using *R*^2^ and adjusted *R*^2^ values.

Additional sensitivity analyses were performed to evaluate whether the association between MAD and PVC burden was independent of prolapse phenotype. The primary multivariable models were sequentially adjusted for bileaflet MVP, Barlow's disease, and both variables simultaneously. To further assess the incremental contribution of MAD relative to baseline PVC burden in predicting postoperative PVC burden, sequential model comparison was performed using nested linear regression models. Changes in model fit (Δ*R*^2^) and partial *R*^2^ values were calculated after sequential addition of log-transformed baseline PVC burden and MAD to a predefined clinical base model.

To assess model stability and potential overfitting given the limited sample size, internal validation of the multivariable models was performed using 5,000 nonparametric bootstrap resamples. The original model specification was refitted in each bootstrap sample. Bootstrap median regression coefficients, percentile-based 95% confidence intervals, and coefficient-direction stability were assessed. Model optimism was additionally estimated by comparing performance in each bootstrap sample with the performance of the same fitted model in the original dataset. Optimism-corrected *R*^2^ and root mean squared error (RMSE) were subsequently calculated.

Statistical analyses were conducted using Python 3.14.0 (Python Software Foundation, Wilmington, DE, USA) with standard scientific libraries. All statistical tests were two-sided, and a *p* value <0.05 was considered statistically significant.

## 3 Results

A total of 77 patients with mitral valve prolapse (MVP) who underwent surgical mitral valve repair and had both pre-operative and post-operative 24-h Holter recordings were included in the final analysis. Mitral annular disjunction (MAD) was present in 36 patients (46.8%) (Table 1). All patients had premature ventricular complexes (PVCs) on the baseline 24-h Holter recording, with preoperative PVC burden ranging from 2,500 to 14,500 beats per 24 h.

**Table 1:** Clinical, echocardiographic, operative and arrhythmic characteristics according to mitral annular disjunction status.

Variable	No MAD (*n* = 41)	MAD present (*n* = 36)	*p* value
Age, years	56.1 ± 7.9; 57.0 (52.0–61.0)	52.1 ± 6.6; 53.0 (47.3–59.0)	0.018
Male sex	17 (41.5%)	13 (36.1%)	0.805
Body-mass index, kg/m^2^	26.2 ± 2.4	23.2 ± 1.2	<0.001
Hypertension	14 (34.1%)	10 (27.8%)	0.722
Diabetes mellitus	5 (12.2%)	7 (19.4%)	0.575
Hyperlipidemia	8 (19.5%)	3 (8.3%)	0.284
COPD	8 (19.5%)	4 (11.1%)	0.484
NYHA class ≥ II	26 (63.4%)	27 (75.0%)	0.396
Hemoglobin	13.45 ± 1.07	13.92 ± 1.25	0.312
Serum creatinine	1.09 ± 0.11	1.10 ± 0.13	0.747
LV ejection fraction, %	63.0 ± 3.1	60.7 ± 4.0	0.006
LV end-diastolic dimension, mm	57.8 ± 3.1	55.9 ± 6.3	0.117
LV end-systolic dimension, mm	36.1 ± 5.7	35.5 ± 4.2	0.645
LA diameter, mm	40.8 ± 3.2	43.8 ± 5.2	0.003
Left atrial volume index, mL/m^2^	38.8 ± 6.3	42.1 ± 8.6	0.056
Pulmonary artery systolic pressure, mmHg	41.0 ± 12.8	38.7 ± 12.1	0.539
Bileaflet MVP	15 (36.6%)	23 (63.9%)	0.031
Barlow's disease	12 (29.3%)	17 (47.2%)	0.166
Pre-op PVC burden/24 h	4,200 [3,200–6,200]	7,800 [7,000–9,575]	<0.001
Post-op PVC burden/24 h	2,200 [1,800–3,500]	5,700 [5,175–6,425]	<0.001
Delta PVC change	−2,300 [−3,000 to −1,100]	−2,450 [−5,275 to 1,675]	0.050
Delta % PVC change	−39.5 ± 29.5	−35.4 ± 20.6	0.113
Pre-op NSVT	18 (43.9%)	21 (58.3%)	0.301
Post-op NSVT	12 (29.3%)	18 (50.0%)	0.104
Post-op Holter time from surgery (months)	8.0 [7.0–9.0]	8.0 [6.0–9.0]	0.295
Post-operative sustained VT detected on Holter monitoring	0 (0.0%)	3 (8.3%)	0.098
Post-operative ventricular bigeminy on Holter monitoring	12 (29.3%)	13 (36.1%)	0.692
Residual MR ≥ moderate	0 (0.0%)	10 (27.8%)	<0.001
Repair strategy			0.643
Leaflet resection	4 (9.8%)	6 (16.7%)	
Blended repair	3 (7.3%)	3 (8.3%)	
Artificial chordae (Non-resection)	34 (82.9%)	27 (75.0%)	
Concomitant tricuspid repair	12 (29.3%)	8 (22.2%)	0.482
Cross-clamp time (min)	142 (118–210)	149 (139.5–204)	0.145
Cardio-pulmonary bypass time (min)	92 (82–138)	85 (70–103.5)	0.033
Hospitalization duration (days)	6 (4–7)	6 (4–6)	0.861
New-onset AF	3 (7.3%)	5 (13.9%)	0.462
Post-operative ICD implantation	0 (0.0%)	3 (8.3%)	0.098
Preoperative beta-blocker use	15 (36.6%)	34 (94.4%)	<0.001
Postoperative beta-blocker use	24 (58.5%)	32 (88.9%)	0.006
Mortality during follow-up	0 (0.0%)	0 (0.0%)	–

MAD, mitral annular disjunction; PVC, premature ventricular complex; NYHA, New York Heart Association Functional Classification; NSVT, nonsustained ventricular tachycardia; MVP, Mitral valve prolapse; VT, ventricular tachycardia; ICD, implantable cardioverter-defibrillator; MR, mitral regurgitation; AF, atrial fibrillation; COPD, chronic obstructive pulmonary disease; BMI, body mass index; LA, left atrial; LV, left ventricular.

Baseline clinical and echocardiographic characteristics according to MAD status are presented in Table 1. Patients with MAD were younger (52.1 ± 6.6 vs. 56.1 ± 7.9 years, *p* = 0.018) and had lower body mass index (23.2 ± 1.2 vs. 26.2 ± 2.4 kg/m^2^, *p* < 0.001). They also demonstrated slightly lower left ventricular ejection fraction (60.7 ± 4.0% vs. 63.0 ± 3.1%, *p* = 0.006) and larger left atrial diameter (43.8 ± 5.2 vs. 40.8 ± 3.2 mm, *p* = 0.003). Bileaflet prolapse was more frequent in the MAD group (63.9% vs. 36.6%, *p* = 0.031).

Operative characteristics are presented in Table 1. All patients underwent mitral valve repair using a Carpentier-Edwards Physio II annuloplasty ring, and no patient underwent concomitant surgical atrial fibrillation ablation (Maze procedure). Repair strategy was similar between groups, with leaflet resection performed in 4 (9.8%) patients without MAD and 6 (16.7%) patients with MAD, blended repair in 3 (7.3%) and 3 (8.3%) patients, and non-resection repair using artificial chordae in 34 (82.9%) and 27 (75.0%) patients, respectively (*p* = 0.643). Concomitant tricuspid valve repair was performed in 12 (29.3%) patients without MAD and 8 (22.2%) patients with MAD (*p* = 0.482). Aortic cross-clamp time was comparable between the no-MAD and MAD groups [median: 142.0 (118.0–210.0) vs. 149.0 (139.5–204.0) min, *p* = 0.145]. Cardiopulmonary bypass time was shorter in the MAD group [median: 85.0 (70.0–103.5) vs. 92.0 (82.0–138.0) min, *p* = 0.033]. Length of hospital stay did not differ between groups [median: 6.0 (4.0–7.0) vs. 6.0 (4.0–6.0) days, *p* = 0.861].

Pre-operative ventricular ectopic burden differed markedly between groups. Patients without MAD had a significantly lower pre-operative PVC burden than those with MAD [4,200 (3,200–6,200) vs. 7,800 (7,000–9,575) PVCs/24 h, Mann–Whitney *U* test, *p* < 0.001]. Although PVC burden decreased following mitral valve repair in both groups, post-operative PVC burden remained significantly lower in patients without MAD than in those with MAD [2,200 (1,800–3,500) vs. 5,700 (5,175–6,425) PVCs/24 h, Mann–Whitney *U* test, *p* < 0.001] (Table 1). In the overall cohort, PVC burden decreased significantly after mitral valve repair, with a median paired change of −2,300 PVCs/24 h (95% CI: −2,700 to −1,800; Wilcoxon signed-rank *p* < 0.001). Significant within-group reductions were observed both in patients without MAD (median change −2,300 PVCs/24 h, 95% CI: −2,700 to −1,700; *p* < 0.001) and in those with MAD (−2,450 PVCs/24 h, 95% CI: −4,000 to −1,800; *p* < 0.001). The between-group comparison of absolute change yielded a Hodges–Lehmann location-shift estimate of −950 PVCs/24 h (MAD minus no MAD; 95% CI: −2,200 to 0; Mann–Whitney *U* test, *p* = 0.050). The relative percentage change was −39.5 ± 29.5% in patients without MAD and −35.4 ± 20.6% in those with MAD; the between-group Hodges–Lehmann estimate was 8.93 percentage points (95% CI: −2.28 to 19.30; Mann–Whitney *U* test, *p* = 0.113).

In the longitudinal repeated-measures analysis of log-transformed PVC burden, PVC burden decreased significantly from preoperative to postoperative assessment in both the no-MAD group (*β* = −0.601, 95% CI: −0.724 to −0.478; *p* < 0.001) and the MAD group (*β* = −0.495, 95% CI: −0.627 to −0.364; *p* < 0.001). However, the time × MAD interaction was not significant (*β* = 0.106, 95% CI: −0.075 to 0.286; *p* = 0.250), providing no evidence that the magnitude of longitudinal change differed according to MAD status.

The prevalence of preoperative NSVT was comparable between patients with and without MAD (58.3% vs. 43.9%, *p* = 0.301). Following mitral valve repair, NSVT remained numerically more frequent in patients with MAD (50.0% vs. 29.3%); however, this difference did not reach statistical significance (*p* = 0.104) (Table 1).

Residual mitral regurgitation ≥ moderate occurred exclusively among MAD-positive patients (27.8% vs. 0%, *p* < 0.001). Incident sustained ventricular tachycardia detected during post-operative Holter monitoring and incident ICD implantation during follow-up were observed exclusively in the MAD group [3 patients (8.3%) each], whereas no deaths occurred during follow-up (Table 1).

### 3.1 Predictors of pre-operative ventricular ectopic burden

In univariate linear regression analysis, preoperative beta-blocker use and mitral annular disjunction (MAD) demonstrated the strongest associations with higher pre-operative PVC burden. Diabetes mellitus, larger left atrial diameter, higher hemoglobin levels, increased left atrial volume index, NYHA functional class ≥ II, and larger left ventricular end-systolic dimension were also significantly associated with increased PVC burden, whereas higher body mass index was associated with lower PVC burden (Table 2).

**Table 2:** Univariate and multivariable linear regression analysis for predictors of pre-operative PVC burden.

Univariate analysis				Multivariable analysis	
Variable	*β coefficient (95% CI)	*p* value	** *R* ^2^	*β coefficient (95% CI)	*p* value
Preoperative beta-blocker use	0.680 (0.531–0.830)	<0.001	0.524	0.363 (0.226–0.501)	<0.001
Mitral annular disjunction	0.660 (0.517–0.803)	<0.001	0.530	0.596 (0.415–0.777)	<0.001
Diabetes mellitus	0.470 (0.204–0.735)	<0.001	0.142	0.272 (0.125–0.420)	<0.001
LA diameter, mm	0.033 (0.011–0.055)	0.003	0.109	−0.012 (−0.026 to 0.003)	0.111
Hemoglobin	0.111 (0.025–0.196)	0.012	0.081	−0.005 (−0.051 to 0.040)	0.821
Left atrial volume index, mL/m^2^	0.016 (0.003–0.029)	0.017	0.073		
NYHA class ≥ II	0.259 (0.043–0.476)	0.019	0.071	−0.039 (−0.159 to 0.081)	0.523
BMI, kg/m^2^	−0.047 (−0.088 to −0.005)	0.029	0.062	0.025 (−0.010 to 0.060)	0.163
LV end-systolic dimension, mm	0.022 (0.001–0.042)	0.037	0.056	0.027 (0.014–0.040)	<0.001
Serum creatinine	0.818 (−0.029 to 1.665)	0.058	0.047		
LV end-diastolic dimension, mm	0.015 (−0.006 to 0.036)	0.163	0.026		
Hypertension	0.138 (−0.084 to 0.360)	0.220	0.020		
Hyperlipidemia	−0.143 (−0.438 to 0.152)	0.338	0.012		
Male sex	0.086 (−0.126 to 0.298)	0.422	0.009		
Age, years	−0.005 (−0.019 to 0.009)	0.517	0.006		
Bileaflet MVP	0.066 (−0.142 to 0.273)	0.530	0.005		
Barlow's disease	0.025 (−0.189 to 0.240)	0.816	0.001		
Pulmonary artery systolic pressure, mmHg	−0.001 (−0.009 to 0.008)	0.830	0.001		
COPD	0.014 (−0.272 to 0.301)	0.921	<0.001		
LV ejection fraction, %	0.000 (−0.028 to 0.029)	0.980	0.000		

MAD, mitral annular disjunction; PVC, premature ventricular complex; NYHA, New York Heart Association Functional Classification; MVP, Mitral valve prolapse; COPD, chronic obstructive pulmonary disease; BMI, body mass index; LA, left atrial; LV, left ventricular.

* β coefficients represent associations with log-transformed pre-operative PVC burden [log(1 + PVC count)].

** *R*^2^ indicates the proportion of variance explained by each univariate model.

In multivariable linear regression analysis, mitral annular disjunction (MAD) remained independently associated with higher pre-operative PVC burden (*β* = 0.596, 95% CI: 0.415–0.777, *p* < 0.001), corresponding to an approximately 81% increase in PVC burden. Preoperative beta-blocker use, diabetes mellitus, and left ventricular end-systolic dimension were also independently associated with higher pre-operative PVC burden (Table 2). Left atrial volume index was not included in the multivariable model because of potential collinearity with left atrial diameter.

### 3.2 Predictors of post-operative ventricular ectopic burden

In univariate linear regression analysis, postoperative beta-blocker use, mitral annular disjunction (MAD), and pre-operative PVC burden demonstrated the strongest associations with higher post-operative PVC burden. Larger left atrial diameter, increased left atrial volume index, residual mitral regurgitation ≥ moderate, diabetes mellitus, higher hemoglobin levels, and larger left ventricular end-systolic dimension were also significantly associated with increased post-operative PVC burden, whereas higher body mass index was associated with lower PVC burden (Table 3).

**Table 3:** Univariate and multivariable linear regression analysis for predictors of post-operative PVC burden.

Variable	*β coefficient (95% CI)	*p* value	** *R* ^2^	*β coefficient (95% CI)	*p* value
Postoperative beta-blocker use	0.814 (0.603–1.024)	<0.001	0.441	0.502 (0.228–0.775)	<0.001
Mitral annular disjunction (MAD)	0.766 (0.586–0.945)	<0.001	0.491	0.743 (0.366–1.120)	<0.001
Log-transformed pre-op PVC burden	0.824 (0.621–1.026)	<0.001	0.466	−0.068 (−0.419 to 0.282)	0.703
LA diameter, mm	0.054 (0.029–0.079)	<0.001	0.199	0.003 (−0.018 to 0.024)	0.791
Left atrial volume index, mL/m^2^	0.030 (0.015–0.045)	<0.001	0.170		
Residual MR ≥ moderate	0.540 (0.188–0.892)	0.003	0.111	−0.045 (−0.369 to 0.280)	0.787
Diabetes mellitus	0.444 (0.114–0.775)	0.009	0.087	0.271 (0.021–0.522)	0.034
Hemoglobin	0.120 (0.016–0.224)	0.024	0.066	−0.026 (−0.090 to 0.039)	0.436
LV end-systolic dimension, mm	0.028 (0.003–0.052)	0.028	0.063	0.011 (−0.022 to 0.044)	0.500
BMI, kg/m^2^	−0.052 (−0.103 to −0.002)	0.042	0.054	0.027 (−0.035 to 0.089)	0.387
Hyperlipidemia	−0.330 (−0.681 to 0.020)	0.064	0.045		
Pulmonary artery systolic pressure, mmHg	0.007 (−0.003 to 0.017)	0.154	0.027		
Bileaflet MVP	0.175 (−0.072 to 0.423)	0.162	0.026		
Serum creatinine	0.675 (−0.361 to 1.710)	0.198	0.022		
Male sex	0.146 (−0.109 to 0.401)	0.257	0.017		
Age, years	−0.009 (−0.026 to 0.008)	0.281	0.016		
NYHA class ≥ II	0.144 (−0.125 to 0.413)	0.288	0.015		
Barlow's disease	0.137 (−0.120 to 0.394)	0.293	0.015		
LV end-diastolic dimension, mm	0.013 (−0.012 to 0.039)	0.299	0.014		
LV ejection fraction, %	−0.005 (−0.039 to 0.029)	0.767	0.001		
COPD	0.047 (−0.298 to 0.393)	0.786	0.001		
Hypertension	0.026 (−0.245 to 0.297)	0.848	<0.001		

MAD, mitral annular disjunction; PVC, premature ventricular complex; NYHA, New York Heart Association Functional Classification; MVP, Mitral valve prolapse; MR, mitral regurgitation; COPD, chronic obstructive pulmonary disease; BMI, body mass index; LA, left atrial; LV, left ventricular.

* *β* coefficients represent associations with log-transformed post-operative PVC burden [log(1 + PVC count)].

** *R*^2^ reflects variance explained by each univariate model.

In multivariable linear regression analysis, MAD remained independently associated with higher post-operative PVC burden. Postoperative beta-blocker use and diabetes mellitus were also independently associated with higher post-operative PVC burden (Table 3). The observed association between beta-blocker use and post-operative PVC burden likely reflects greater baseline arrhythmic burden and symptom severity among patients requiring medical therapy, rather than a direct treatment effect. Notably, pre-operative PVC burden was not independently associated with post-operative PVC burden after adjustment (*p* = 0.703).

### 3.3 Sensitivity analyses

Sensitivity analyses were performed to evaluate the relative contributions of mitral annular disjunction (MAD) and baseline arrhythmic burden to post-operative PVC burden (Table 4). In the model excluding pre-operative PVC burden (Model 1), MAD remained independently associated with higher post-operative PVC burden. Postoperative beta-blocker use and diabetes mellitus were also independently associated with higher PVC burden. In the model excluding MAD (Model 2), pre-operative PVC burden was significantly associated with post-operative PVC burden.

**Table 4:** Sensitivity analyses for predictors of post-operative PVC burden.

Variable	Model 1 (Without pre-op PVC) *β (95% CI)	*p* value	Model 2 (Without MAD) *β (95% CI)	*p* value
Postoperative beta-blocker	0.492 (0.221–0.763)	<0.001	0.494 (0.235–0.753)	<0.001
Mitral annular disjunction (MAD)	0.690 (0.399–0.982)	<0.001	—	—
Log pre-op PVC burden	—	—	0.376 (0.066–0.686)	0.017
LA diameter, mm	0.004 (−0.016 to 0.024)	0.696	0.023 (0.000–0.046)	0.050
Residual MR ≥ moderate	−0.042 (−0.360 to 0.277)	0.798	0.108 (−0.189 to 0.404)	0.478
Diabetes mellitus	0.247 (0.012–0.481)	0.039	0.115 (−0.126 to 0.356)	0.350
Hemoglobin	−0.024 (−0.087 to 0.039)	0.456	0.007 (−0.070 to 0.084)	0.850
LV end-systolic dimension, mm	0.010 (−0.019 to 0.039)	0.506	0.005 (−0.031 to 0.040)	0.797
BMI, kg/m^2^	0.025 (−0.039 to 0.090)	0.445	−0.044 (−0.102 to 0.015)	0.145

MAD, mitral annular disjunction; PVC, premature ventricular complex; MR, mitral regurgitation; BMI, body mass index; LV, left ventricular.

* *β* coefficients represent associations with log-transformed post-operative PVC burden [log(1 + PVC count)].

Additional sensitivity analyses were performed by sequentially adjusting the multivariable models for bileaflet MVP, Barlow's disease, and both variables simultaneously. In the pre-operative PVC model, the association between MAD and PVC burden remained statistically significant after adjustment for bileaflet MVP (*β* = 0.627, 95% CI: 0.419–0.834; *p* < 0.001), Barlow's disease (*β* = 0.615, 95% CI: 0.425–0.804; *p* < 0.001), and both variables simultaneously (*β* = 0.614, 95% CI: 0.397–0.832; *p* < 0.001). The estimated regression coefficient for MAD changed only minimally compared with the primary model (*β* = 0.596, 95% CI: 0.415–0.777; *p* < 0.001).

Similarly, in the post-operative PVC model, MAD remained independently associated with higher PVC burden after adjustment for bileaflet MVP (*β* = 0.662, 95% CI: 0.196–1.128; *p* = 0.005), Barlow's disease (*β* = 0.716, 95% CI: 0.301–1.132; *p* < 0.001), and both variables simultaneously (*β* = 0.654, 95% CI: 0.187–1.120; *p* = 0.006). Although the estimated effect of MAD remained directionally consistent after additional adjustment for bileaflet MVP and/or Barlow's disease, the confidence intervals became wider, reflecting reduced precision after accounting for correlated phenotypic variables. Nevertheless, the association remained statistically significant across all adjusted models.

Multicollinearity was assessed using VIFs. In the preoperative models, VIF values ranged from 1.11 to 4.51, with the highest values observed for bileaflet MVP (VIF = 4.51), MAD (VIF = 4.15), and Barlow's disease (VIF = 3.28) in the fully adjusted phenotype model. In the postoperative models, VIF values ranged from 1.16 to 6.80, with the highest values observed for MAD (VIF = 6.80), bileaflet MVP (VIF = 3.90), and Barlow's disease (VIF = 3.07). Although moderate collinearity was observed between MAD and MVP phenotypic variables in the postoperative phenotype-adjusted models, VIF values remained below 7, and no evidence of severe multicollinearity was identified.

For post-operative PVC burden, an additional nested-model analysis was performed using a clinical base model that included postoperative beta-blocker use, diabetes mellitus, left atrial diameter, residual moderate or greater mitral regurgitation, hemoglobin, left ventricular end-systolic dimension, body mass index, bileaflet MVP, and Barlow's disease. In this analysis, MAD was added to the clinical base model to assess whether its association with postoperative PVC burden remained evident after accounting for these clinical and phenotypic covariates. Addition of MAD increased the model *R*^2^ by 0.083. In a further model that additionally accounted for log-transformed baseline PVC burden, the association with MAD remained statistically significant (Δ*R*^2^ = 0.053, partial *R*^2^ = 0.177, *p* < 0.001). These analyses were performed as sensitivity analyses to assess the consistency of the observed association with MAD across complementary model specifications and should not be interpreted as establishing its relative predictive importance compared with other covariates.

In a sensitivity analysis restricted to patients with successful mitral valve repair, defined as residual MR less than moderate (*n* = 67; 41 without MAD and 26 with MAD), longitudinal PVC burden was additionally analyzed after accounting for beta-blocker use as a time-varying covariate. PVC burden decreased significantly in both the no-MAD group (*β* = −0.705, 95% CI: −0.836 to −0.574; *p* < 0.001) and the MAD group (*β* = −0.456, 95% CI: −0.538 to −0.374; *p* < 0.001). In this restricted analysis, the time × MAD interaction was significant (*β* = 0.249, 95% CI: 0.094–0.404; *p* = 0.002), indicating a less pronounced longitudinal reduction in PVC burden among patients with MAD.

### 3.4 Bootstrap internal validation

Bootstrap internal validation with 5,000 resamples demonstrated stability of the principal regression estimates. For MAD, the bootstrap median coefficients were 0.611 (95% CI: 0.430–0.810) in the preoperative model, 0.757 (95% CI: 0.381–1.102) in the postoperative primary model, and 0.689 (95% CI: 0.394–0.953) in postoperative sensitivity model 1. The MAD coefficient remained positive in 100%, 99.98%, and 100% of bootstrap resamples, respectively. After correction for bootstrap-estimated optimism, *R*^2^ decreased from 0.782 to 0.731 for the preoperative model, from 0.748 to 0.668 for the postoperative primary model, from 0.747 to 0.678 for sensitivity model 1, and from 0.670 to 0.586 for sensitivity model 2.

## 4 Discussion

In this retrospective cohort of patients undergoing mitral valve repair for severe mitral regurgitation secondary to mitral valve prolapse, a substantial ventricular arrhythmic burden was observed before surgery and remained detectable during follow-up despite a significant reduction in overall premature ventricular ectopy after surgical repair. Nearly half of the cohort had echocardiographically identified mitral annular disjunction (MAD), and these patients exhibited a substantially higher baseline and postoperative ventricular ectopy. These findings highlight the complex interplay between structural mitral valve abnormalities and ventricular arrhythmogenesis in MVP.

Mitral valve prolapse has increasingly been recognized as a complex arrhythmogenic syndrome rather than a purely valvular disorder. Several studies have described a distinct high-risk phenotype characterized by bileaflet prolapse, mitral annular disjunction (MAD), papillary muscle fibrosis, and frequent ventricular ectopy (16, 17). In our cohort, preoperative Holter monitoring demonstrated a high burden of PVCs and episodes of nonsustained ventricular tachycardia, findings that are consistent with previous reports in patients with arrhythmogenic MVP. The particularly high ectopic burden observed among MAD-positive patients further supports the concept that annular–ventricular dissociation may contribute to electrical instability.

The observed association between beta-blocker use and higher PVC burden should be interpreted cautiously, as it likely reflects confounding by indication, whereby patients with more severe baseline arrhythmic profiles were more likely to receive medical therapy rather than beta-blockers exerting a direct pro-arrhythmic effect.

Diabetes mellitus was also independently associated with higher pre-operative and post-operative PVC burden in our cohort. Although this finding should be interpreted cautiously given the observational design and limited sample size, metabolic and microvascular alterations associated with diabetes may plausibly contribute to myocardial electrical instability and increased ventricular ectopy (18). Previous studies have similarly suggested a potential association between diabetes-related myocardial remodeling and arrhythmic susceptibility (19). However, the precise mechanisms underlying this relationship in patients with mitral valve prolapse remain uncertain and warrant further investigation.

The mechanisms linking MAD to ventricular arrhythmias are likely multifactorial. Mechanical systolic separation between the mitral annulus and the ventricular myocardium may increase traction forces on the papillary muscles and the basal inferolateral myocardium, promoting localized myocardial stretch, fibrosis, and electrical heterogeneity (7, 8). These structural and mechanical alterations may create a substrate that predisposes to ventricular ectopy and reentrant arrhythmias. Cardiac magnetic resonance imaging studies have further demonstrated an association between MAD and myocardial fibrosis, supporting the role of structural remodeling in arrhythmogenesis (9, 15).

The interpretation of MAD has evolved considerably in recent years. Rather than representing a discrete pathological substrate, contemporary multimodality imaging studies suggest that MAD encompasses a spectrum of anatomical variants, many of which may represent normal anatomical morphology. Emerging evidence indicates that arrhythmic risk is primarily associated with inferolateral disjunction accompanied by prolapse-related myocardial remodeling, fibrosis, and systolic curling, whereas anterior or inferior disjunction may simply reflect physiological anatomical variation (10, 11). Because circumferential localization was not available in our study, the echocardiographic diagnosis of MAD should be interpreted as an imaging marker identified on two-dimensional parasternal long-axis imaging rather than a definitive characterization of the arrhythmogenic phenotype. Therefore, our findings should not be interpreted as demonstrating that the mere presence of MAD is independently arrhythmogenic. Rather, they indicate that patients classified as having MAD on routine echocardiography exhibited a higher postoperative ventricular arrhythmic burden, while acknowledging that the underlying anatomical phenotype could not be fully characterized.

Mitral valve repair remains the preferred treatment for severe degenerative mitral regurgitation, primarily because of its favorable effects on survival and preservation of left ventricular function (20). In addition to improving hemodynamics, surgical repair may reduce abnormal mechanical stress on the mitral apparatus and papillary muscles, thereby potentially mitigating arrhythmogenic triggers (21). In the present study, overall PVC burden decreased significantly after surgery, suggesting that correction of mitral regurgitation and restoration of valve competence may partially alleviate arrhythmogenic stimuli related to volume overload and abnormal leaflet motion. While cardiopulmonary bypass time differed statistically between groups (median 92 vs. 85 min, *p* = 0.033), the absolute difference of 7 min is within routine variability of the operation and is unlikely to carry clinical meaning at this sample size. Of note, cross-clamp time, a closer surrogate for operative complexity, did not differ between groups (*p* = 0.145). Neither variable should be interpreted as indicating a difference in procedural complexity.

However, the reduction in ventricular ectopy was not uniform across the study population. Patients with preoperative MAD continued to exhibit a substantially higher postoperative arrhythmic burden following mitral valve repair. These findings suggest that correction of mitral regurgitation alone may not completely eliminate the structural and mechanical factors associated with ventricular arrhythmogenesis in patients with MAD. Experimental and clinical evidence suggests that annular-ventricular abnormalities may impose abnormal mechanical stress on the papillary muscles and adjacent myocardium, thereby contributing to an arrhythmogenic substrate (22). However, because postoperative MAD was not reassessed in the present study, we cannot determine whether the observed postoperative arrhythmic burden reflects persistence of MAD itself, residual structural abnormalities, or other features of advanced degenerative mitral valve disease.

These observations are consistent with emerging evidence from prior studies. Karin Lodin et al. demonstrated that both the presence and extent of MAD were independently associated with ventricular arrhythmias following mitral valve surgery (22). Likewise, accumulating evidence suggests that structural myocardial abnormalities, including papillary muscle fibrosis and annular disjunction, may represent an arrhythmogenic substrate that is not fully reversed by surgical intervention (7–9, 22).

Our findings extend previous observations regarding the effect of mitral valve surgery on ventricular arrhythmias in MVP. Naksuk et al. (23) demonstrated that ventricular ectopy does not uniformly decrease after mitral valve surgery in patients with bileaflet MVP, while Ascione et al. (24) similarly reported heterogeneous arrhythmic responses after repair in patients with Barlow's disease. More recently, Ertugay et al. (25) demonstrated a reduction in ventricular ectopic burden after surgery in patients with MAD and suggested that the extent of MAD may influence the magnitude of this reduction. Lodin et al. (22), in a substantially larger cohort, showed that preoperative MAD remained associated with an increased long-term risk of ventricular arrhythmias despite surgical correction of the anatomical disjunction. Our study complements these reports by directly comparing patients with and without preoperative MAD using paired quantitative 24-h PVC measurements before and after mitral valve repair. This design allowed us to evaluate not only postoperative arrhythmic burden but also within-patient longitudinal changes and whether these changes differed according to MAD status. Collectively, our findings support the concept that mitral valve repair may reduce ventricular ectopy while also suggesting that preoperative MAD identifies a subgroup with greater postoperative arrhythmic burden, although the magnitude of longitudinal change according to MAD status appears sensitive to the analytic approach and should not be interpreted as evidence of a causal effect of MAD.

In complementary sensitivity analyses, the association between MAD and postoperative PVC burden remained consistent across alternative model specifications, including models accounting for baseline preoperative PVC burden and prolapse phenotype. These findings support the robustness of the observed association across different model specifications but should not be interpreted as establishing the relative predictive importance of MAD compared with other covariates.

It is noteworthy that neither bileaflet MVP nor Barlow's disease was significantly associated with ventricular ectopic burden in the present cohort. Given the relatively small sample size and the use of two-dimensional echocardiography for phenotypic classification, these findings should not be interpreted as evidence against the established association between MVP phenotype and ventricular arrhythmias. Rather, limited statistical power and potential phenotype misclassification may have attenuated these relationships.

From a clinical perspective, these findings underscore the importance of comprehensive rhythm assessment in patients with MVP, particularly in the presence of preoperative MAD. Identification of patients with a higher postoperative ventricular ectopic burden may help guide postoperative surveillance strategies, including extended rhythm monitoring and closer clinical follow-up. Although sustained ventricular tachycardia and implantable cardioverter-defibrillator implantation were observed only among patients with MAD in our cohort, these events were infrequent and did not differ significantly between groups. Therefore, these findings should be considered exploratory and hypothesis-generating rather than evidence of a distinct high-risk arrhythmogenic phenotype. Given that sudden cardiac death remains relatively uncommon in patients with MVP, further prospective studies are needed to determine whether specific anatomical phenotypes, including MAD, identify patients who may benefit from more intensive postoperative arrhythmic surveillance (4, 6, 7, 19).

In addition, the observation that residual mitral regurgitation occurred exclusively among MAD-positive patients may suggest structural instability at the annular-ventricular interface. Although the clinical implications of this finding remain uncertain, it may reflect ongoing geometric and mechanical abnormalities contributing to sustained myocardial stress and arrhythmogenic susceptibility.

The sensitivity analysis provides additional context for these observations. Although the time × MAD interaction was not significant in the overall cohort, it became significant when the analysis was restricted to patients with successful repair and beta-blocker use was accounted for as a time-varying covariate, with a less pronounced reduction in PVC burden observed in patients with MAD. This finding should be interpreted cautiously. The substantial imbalance in beta-blocker use between groups, the observational nature of treatment allocation, and the smaller sample size of the restricted cohort limit causal interpretation. Accordingly, this analysis should be considered exploratory and does not establish that MAD itself accounts for the differential postoperative change in PVC burden.

The relatively high proportion of patients with residual moderate or greater MR also warrants consideration. Although residual MR showed a significant association with postoperative PVC burden in univariable analysis, this association was no longer significant after adjustment for other clinical and echocardiographic variables. This attenuation may reflect interrelationships among markers of underlying valve pathology and postoperative valve function rather than an independent effect of residual MR itself. In addition, as this was a retrospective observational study, the contribution of surgical technique and repair durability cannot be completely separated from patient-related structural characteristics.

It should be acknowledged that inclusion in the present study required the availability of both preoperative and postoperative 24-h Holter recordings. Therefore, the study cohort likely represents a subset of patients who underwent more intensive rhythm surveillance in routine clinical practice, probably because of clinically recognized ventricular ectopy before surgery. This should be considered when interpreting our findings, as the observed associations may not be fully applicable to unselected populations of patients undergoing mitral valve repair.

Finally, although NSVT appeared to decrease following surgery, this reduction did not reach statistical significance, likely reflecting the limited sample size and the inherent limitations of 24-h Holter monitoring. More prolonged and systematic rhythm surveillance may provide a more comprehensive assessment of residual postoperative arrhythmic risk.

Overall, these findings suggest that mitral valve repair is associated with a meaningful reduction in ventricular ectopic burden in patients with MVP. However, patients with preoperative MAD exhibited a substantially higher postoperative PVC burden despite experiencing a proportional reduction in ectopy comparable to that of patients without MAD. These findings are consistent with preoperative MAD identifying a subgroup of patients at higher postoperative arrhythmic risk. Accordingly, these results support the potential value of continued postoperative rhythm surveillance in patients with preoperative MAD.

### 4.1 Limitations

This study has several limitations. First, this was a retrospective single-center analysis with a relatively small sample size, which may limit statistical power and generalizability, particularly for subgroup analyses. Second, arrhythmic burden was assessed using 24-h Holter monitoring only, potentially underestimating intermittent or day-to-day variability in ventricular arrhythmias. Furthermore, although all patients with a previously documented diagnosis of atrial fibrillation were excluded, clinically silent or previously undiagnosed paroxysmal atrial fibrillation could not be completely ruled out because of the retrospective study design.

Third, cardiac magnetic resonance imaging was not systematically available, precluding detailed assessment of myocardial fibrosis and other structural arrhythmogenic substrates. Assessment was based exclusively on two-dimensional parasternal long-axis transthoracic echocardiography, precluding reliable assessment of circumferential location and extent of annular disjunction. Consequently, we were unable to distinguish common anatomical variants from the inferolateral phenotype increasingly recognized as the arrhythmogenic form of MAD by cardiac CT and CMR. Therefore, our findings should be interpreted as associations with echocardiographically detected MAD rather than with the contemporary imaging-defined arrhythmogenic MAD phenotype. In addition, MAD was assessed retrospectively using two-dimensional echocardiography without formal interobserver or intra-observer variability analysis; therefore, some degree of measurement variability cannot be excluded.

Another important limitation is that MAD was assessed only preoperatively. Because surgical mitral valve repair, particularly ring annuloplasty and annular plication techniques, may substantially modify or even eliminate mitral annular disjunction, we could not determine the postoperative persistence or resolution of MAD. Therefore, our analyses relate postoperative ventricular ectopy to the baseline anatomical phenotype rather than to the postoperative substrate. Future prospective studies incorporating standardized serial echocardiographic or cardiac magnetic resonance imaging assessment of MAD before and after surgery are needed to clarify whether surgical correction of MAD mediates the observed changes in ventricular arrhythmic burden. Although operative variables were retrieved from the archived surgical records, annuloplasty ring size was not consistently documented and therefore could not be reliably analyzed.

Finally, the observational design precludes causal inference, and the association between beta-blocker use and arrhythmic burden likely reflects confounding by indication, whereby patients with more severe arrhythmic profiles were more likely to receive medical therapy. Furthermore, because structural disease severity, prolapse phenotype, and treatment decisions are closely interrelated, residual confounding cannot be completely excluded despite multivariable adjustment and sensitivity analyses. Nevertheless, the association between preoperative MAD and postoperative arrhythmic burden remained consistent across multiple complementary analyses, supporting the robustness of the observed association within the limitations of this observational study.

## 5 Conclusion

Mitral valve prolapse is associated with a substantial burden of ventricular arrhythmias that decreases following mitral valve repair. In this retrospective cohort, surgical correction of severe mitral regurgitation was associated with a significant reduction in premature ventricular ectopic burden. However, patients with preoperative mitral annular disjunction (MAD) exhibited a higher PVC burden both before and after surgery, despite experiencing a proportional reduction in ectopy comparable to that observed in patients without MAD. These findings suggest that echocardiographically detected preoperative MAD identifies a subgroup of patients at increased risk of a higher postoperative ventricular arrhythmic burden. Given the observational design, the absence of postoperative MAD reassessment, and the evolving understanding of MAD, these findings should be interpreted as an association rather than evidence of causality. Larger prospective studies incorporating multimodality imaging, standardized postoperative assessment of MAD, and extended rhythm monitoring are warranted to refine postoperative risk stratification and clarify the mechanistic relationship between MAD and ventricular arrhythmias after mitral valve repair.

## Data Availability

The data analyzed in this study is subject to the following licenses/restrictions: The data that support the findings of this study are available from the corresponding author upon reasonable request. Requests to access these datasets should be directed to drumeyirsavur@hotmail.com.
